# Long-term outcomes in leucine-rich glioma inactivated-1 autoimmune encephalitis and associated biomarkers of inflammation and neuronal and glial injury

**DOI:** 10.3389/fneur.2025.1583892

**Published:** 2025-05-21

**Authors:** Tyler L. Borko, Phillip Winters, Kelli M. Money, Stefan Sillau, Sadie Eggmann, Sean Selva, Alanna Ritchie, Ryan Kammeyer, Gregory P. Owens, Jeffrey L. Bennett, Amanda L. Piquet

**Affiliations:** ^1^Midwestern University Arizona College of Osteopathic Medicine, Glendale, AZ, United States; ^2^Department of Neurology, University of Colorado Anschutz Medical Campus, Aurora, CO, United States; ^3^Department of Pediatrics and Neurology, Children’s Hospital Colorado Anschutz Medical Campus, Aurora, CO, United States; ^4^Department of Ophthalmology, Programs in Neuroscience and Immunology, University of Colorado Anschutz Medical Campus, Aurora, CO, United States

**Keywords:** autoimmune encephalitis, leucine-rich glioma inactivated 1, biomarkers, neurofilament light chain, glial fibrillary acidic protein

## Abstract

**Introduction:**

Leucine-rich glioma inactivated 1 (LGI1) autoimmune encephalitis (AE) is characterized by seizures, as well as cognitive, memory, and behavioral disturbances. Blood-based biomarkers for inflammation and neuronal and glial injury have been evaluated as potential markers of disease severity and prognosis in AE.

**Methods:**

Patients diagnosed with LGI1 AE, confirmed by cell-based assay, were enrolled and followed prospectively to gather plasma samples for biomarker testing. Biomarkers of neuronal and glial injury included plasma neurofilament light chain (NfL), glial fibrillary acidic protein (GFAP), ubiquitin c-terminal hydrolase L1 (UCHL-1), tau, and cytokine markers of inflammation. Biomarker data were logarithmically transformed and analyzed using longitudinal regression with the ratio of means between LGI1 AE and non-inflammatory controls. Clinical data were collected and correlated with blood-based biomarkers to assess their relationship to disease severity and long-term outcomes.

**Results:**

Twenty-one LGI1 AE patients were enrolled from October 2018 to April 2024, and 16 migraine headache patients (56.3% male; average age: 58 years) served as non-inflammatory controls. One LGI1 AE patient in our cohort had a clinical relapse. Modified Rankin Score (mRS) and Montreal Cognitive Assessment (MoCA) improved over time. The mRS at symptom onset was 3.34 and dropped to 0.56 in a 5-year follow-up. Mean MoCA scores were 18.45 at the onset and increased to 29.40 in the 6-year follow-up. The model estimated geometric mean plasma NfL values at disease diagnosis to be 11.86 pg/mL; it was estimated to be 6.07 pg/mL when compared to non-inflammatory controls. The model also estimated the plasma GFAP values to be 77.70 pg/mL; it was estimated to be 36.26 pg/mL when compared to non-inflammatory controls. The trend of clinical improvement is paralleled with a slow decline in NfL and GFAP levels, returning to levels like our control population after 6 and 3 years, respectively. MoCA scores tended to recover more quickly in patients presenting with lower Nfl scores at symptom onset.

**Conclusion:**

Improved clinical symptoms were correlated with improvements in initially high NfL and GFAP levels. In one patient with a clinical relapse, NfL and GFAP levels increased. NfL and GFAP may be useful biomarkers of disease progression in patients with LGI1 AE. However, additional studies are needed to better understand the effects of immunotherapy.

## Introduction

Anti-leucine-rich glioma inactivated-1 (LGI1) autoimmune encephalitis (AE) is a type of AE characterized by seizures and cognitive, behavioral, and memory disturbances. LGI1 AE typically presents with faciobrachial dystonic seizures (FBDS) or other types of focal seizures, followed by memory impairment. Although FBDS are pathognomonic for LGI1 AE, these can go unrecognized, and not all patients present with pathognomonic seizure activity (1, 2). First-line immunotherapy for LGI1 AE is corticosteroids; patients usually show a significant positive response, but some require additional treatments such as intravenous immunoglobulin (IVIg) or plasma exchange (PLEX) (3). This is typically followed by second-line therapies of variable duration, including anti-CD20 therapy, such as rituximab, and less commonly oral immunosuppression such as mycophenolate mofetil (MMF), but alternate therapies like satralizumab are actively being investigated (4). The majority of patients treated with immunotherapy become seizure-free following the initiation of immunotherapy, usually within a year (4). While cognitive symptoms tend to improve after immunotherapy, mild residual cognitive impairment and amnesia of the acute disease period frequently persist (2). Relapses are common, occurring in approximately 35% of patients (2).

Serum or plasma neurofilament light (NfL) is a neuron-specific cytoskeletal protein released from neurons after neuroaxonal injury (5), and serum or plasma glial fibrillary acidic protein (GFAP) is an astrocytic structural protein released with glial cell damage or microglial activation (6). Cerebrospinal fluid (CSF) and plasma biomarkers have been adapted to monitor disease activity and severity in individuals with neurodegenerative and neuroinflammatory diseases (7–11). For example, higher serum glial fibrillary acidic protein (GFAP) levels correlate with poor memory function and white matter health, independent of markers for AD pathology (12, 13).

Autoimmune encephalitides, including LGI1 AE, may be associated with transient brain tissue damage, and markers of neuronal damage have been explored as potential biomarkers of disease activity. Studies are limited and particularly focused on NfL. Elevated CSF NfL at the time of AE diagnosis has been shown to correlate with disability at 1 year (14) and worse long-term disease outcomes in *N*-methyl-d-aspartate receptor (15) and LGI1 AE (16). Serum NfL elevations in anti-NMDAR encephalitis have also been shown to be a good predictor of functional status at 1 year (17) and serum NfL elevation may help differentiate between anti-NMDAR encephalitis and primary psychiatric psychosis (18); however, additional studies looking at longer-term outcomes are sparse (19). To date, similar data in LGI1 AE is lacking.

Cytokines, interleukin (IL)-6, IL-17, and IL-10, have been implicated in AE pathogenesis. Several studies have explored the CSF cytokine profile of various autoimmune encephalitides, but their interpretation has been limited due to small sample size and high heterogeneity. Elevated levels of intrathecal IL-6 and IL-17A were associated with worse clinical status, clinical severity, relapse, and outcomes (23). Data on IL-6 and other cytokine markers in LGI1 AE are sparse in CSF and plasma (16).

We enrolled LGI1 AE patients in a longitudinal study to correlate clinical outcome measures with plasma and CSF biomarkers. We analyzed both cross-sectional and longitudinal NfL, GFAP, UCH-L1, and cytokine markers and evaluated their association with measures of disability (Modified Rankin scale; mRS), cognitive function (Montreal Cognitive Assessment; MoCA), and LGI1-specific clinical measures of disease activity, including seizure frequency. Our study aimed to understand the relationship between blood-based biomarkers of neuronal and glial injury and clinical measures of long-term disability and cognitive function. As an exploratory measure in this study, we also analyzed cytokine markers of inflammation at disease onset.

## Materials and methods

### Standard protocol approvals, registrations, and patient consents

This study was approved by the Colorado Multiple Institutional Review Board for secondary use (COMIRB: 18–1,361 and 12–0968). All LGI1 AE participants or their legally authorized representative gave written informed consent to participate in the study during data collection.

### Patients

We prospectively enrolled 21 University of Colorado Hospital patients on the inpatient neurology service or through the outpatient Autoimmune Neurology and Neuroimmunology clinics from October 2018 to April 2024 under IRB #18–1,361. All participants were diagnosed with LGI1 AE, confirmed by a positive anti-LGI1 antibody in the blood or both blood and CSF on cell-based assay (CBA) at Mayo Clinic Laboratories, and met the criteria proposed by Graus and colleagues in 2016 for autoimmune encephalitis (20). Patients were excluded if they did not meet criteria for autoimmune encephalitis (e.g., one patient was excluded from the analysis due to having a peripheral nerve hyperexcitability syndrome in the setting of LGI1 antibody positivity). Clinical data were collected from their clinic visit by an autoimmune neurologist (ALP). They included dates of diagnosis and symptom onset, results of CSF studies, MoCA testing, mRS, and symptoms including FBDS, focal or generalized seizures, memory changes, and subjective report of personality changes from caregivers. First-line and second-line therapies were documented along with results of electroencephalogram (EEG), magnetic resonance imaging (MRI), and positron emission tomography (PET) when available. Follow-up clinical assessments, including MoCA, were obtained at standard of care follow-ups approximately every 3–6 months. MoCA was performed at the clinic visit by the neurologist (ALP, KMM, and RK) or trained nursing staff during their standard of care in-person visit per our typical clinic protocols. Modified Rankin Scale was not collected prospectively through designated questionnaires at each visit. Still, it was determined retrospectively through additional chart review based on clinical data collected (mRS determined by TLB and ALP). To assess clinical progression and recovery, mRS and MoCA were evaluated up to 6 years from symptom onset. Blood and CSF were collected at hospital and outpatient clinic visits as part of the prospective study and stored in our Autoimmune, Paraneoplastic, and Inflammatory Neurological Disease Registry at the University of Colorado.

### Controls

We utilized 16 non-inflammatory control patients from the RMMSC Biorepository for the Study of Neuroimmunological Disorders (IRB #12–0968). Control patients were identified to have a typical migraine or chronic daily headache syndrome and no history of any autoimmune disease or prior history of meningitis or encephalitis. Patients were excluded as migraine headache controls if they had any of the following features: (1) Evidence of idiopathic intracranial hypertension (defined by an opening pressure ≥ 25 cm H_2_O), (2) inflammatory CSF demonstrated by a white blood cell count WBC ≥ 5, the presence of unique oligoclonal bands, and protein count ≥ 45, (3) history of autoimmune disease, or (4) a known neurodegenerative or neurocognitive disorder.

### Sample processing and storage

Blood was collected in sodium citrate tubes, centrifuged, and then supernatant (plasma) aliquoted at room temperature and stored at −80°C.

### Biomarkers

Plasma NfL, GFAP, UCHL-1, and tau were tested in duplicate using the SIMOA 4-Plex A kit (Quanterix SR-X by SIMOA platform, Billerica, USA). A 10-panel cytokine array (IL-12p70, IL-1β, IL-4, IL-5, interferon-gamma [IFNγ], IL-6, IL-8, IL-22, tumor necrosis factor-alpha [TNFα], IL-10) was tested using the SIMOA COREPLEX planar array kit (Quanterix SP-X by SIMOA platform, Billerica, MA, USA).

### Statistical analysis

**Figure 1:**
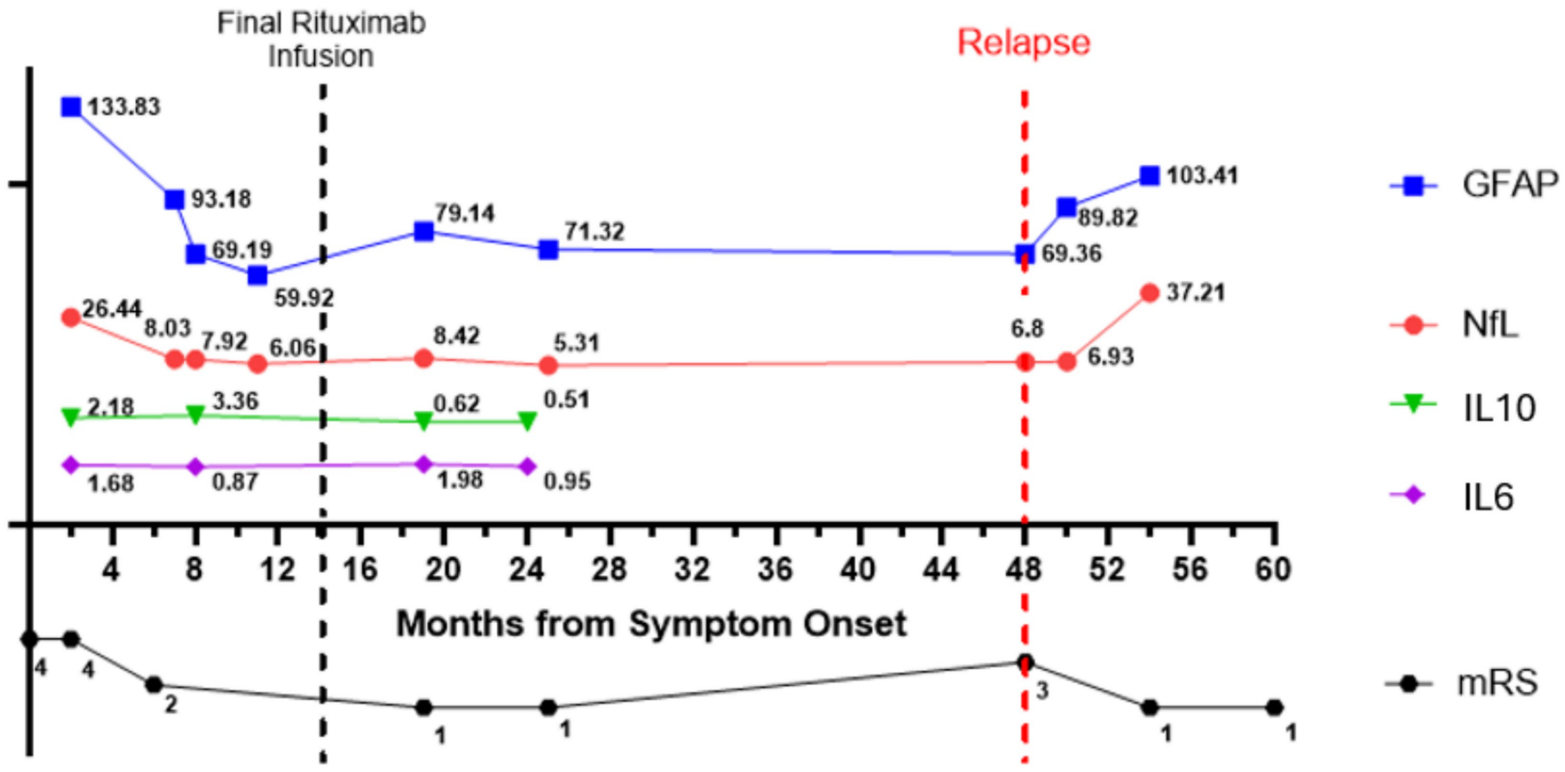
At the time of initial diagnosis, values for neurofilament light chain (NfL) and glial fibrillary acidic protein (GFAP) were 26.44 and 133.83 pg/mL, respectively, and decreased to 71.32 and 5.31 pg/mL, respectively, at 2 years post-diagnosis. Cytokine levels for IL-6 and IL-10 dropped from 1.68 and 2.18, respectively, to 0.95 and 0.51 at 6 months post-diagnosis. Similarly, neurological disability as measured by mRS, improved throughout this time course, with an initial mRS score of 4 at diagnosis, followed by improvement down to 1 after 2 years. The patient had a clinical relapse with return of faciobrachial dystonic seizures at 4 years post-diagnosis and 3 years after their last rituximab infusion. At the time of the relapse, steroids, rituximab, and lacosamide were restarted. At the time of the relapse, there was an increase in the mRS to 3, correlating to an increase in plasma NFL and GFAP.

Summary statistics were obtained during initial collection for LGI1 patient demographics, disease characteristics, diagnostic testing, and therapies. Demographics were compared between LGI1 AE and non-inflammatory control groups with chi-squared and Fisher’s exact tests for categorical variables, and with a *t*-test for continuous variables. Plasma biomarkers of inflammation and neuronal and glial injury concentrations were logarithmically transformed because of skew, and outcomes were analyzed using longitudinal regression, with slopes for disease duration and age. The slopes were estimated and tested, and comparisons between LGI1 AE patients and controls were made where applicable. When back-transforming from logarithmic to original scale, log means become geometric means, log mean differences become ratios of geometric means, and log linear relationships become exponential relationships. Scatter plots with fitted regression were created. Longitudinal regression was also used to estimate and test the time slopes for mRS and MoCA, and to investigate the effects of biomarkers on mRS and MoCA. Specifically, initial measured NfL and GFAP (using log scale) concentrations were tested as effect modifiers on the time slopes for mRS and MoCA, and time-varying NfL and GFAP (using log scale) were tested for association with mRS and MoCA. When combining measurements on different variables with different dates, the nearest date was selected as a match, provided it was within 30 days. Initial biomarker measures (log scale) were also compared between AE and controls with two-sample *t*-tests. A *p*-value below or equal to 0.05 was used for statistical significance. Statistical analyses were performed on an available case basis, using SAS 9.4, and plots were created using STATA 17.0 (College Station, TX, USA). Figure 1 was created using Prism (version 10.4.0).

### Correlation analysis of plasma NfL and GFAP with clinical outcome

Initial NfL and GFAP levels at the onset of disease were used to analyze the estimated change in both MoCA and mRS to assess whether initial elevations in each biomarker impacted long-term outcomes. Time-varying NfL and GFAP were tested for synchronous association with MoCA and mRS (closest match within 30 days). Time-varying NfL and GFAP were tested for synchronous association with MoCA and mRS (closest match within 30 days). For matching NfL and GFAP to the MoCA data, there were 20 usable observations on 13 unique patients (Table 1). To match the NfL and GFAP to the mRS data, there were 30 usable observations on 19 unique patients (Table 2).

**Table 1:** Plasma NfL and GFAP association with change in Montreal Cognitive Assessment (MoCA).

Intentionally	Doubling initial plasma NfL	95% CI (units); *p*-value	Doubling initial plasma GFAP	95% CI (units); *p*-value
Baseline change	1.4556 units	(−2.8657, 5.7769); *p* = 0.4785	1.4981 units	(−3.8240, 6.8201); *p* = 0.5516
Disease duration slope change	−1.6760 units/year	(−4.6996, 1.3476); *p* = 0.2611	−0.7545 units	(−3.2826, 1.7736); *p* = 0.5354

	Doubling synchronous plasma NfL (units)	95% CI (units); *p*-value	Doubling synchronous plasma GFAP	95% CI (units); *p*-value
Unadjusted	0.49	(−1.23, 2.22); *p* = 0.5204	0.26 units	(−1.49, 2.01); *p* = 0.7430
Adjusted for time-varying age	0.72	(−1.01, 2.44); *p* = 0.3532	0.64 units	(−1.25, 2.52); *p* = 0.4502
Adjusted for baseline age and time-varying disease duration	0.91	(−0.97, 2.78); *p* = 0.2940	0.56 units	(−1.49, 2.60); *p* = 0.5380

**Table 2:** Plasma NfL and GFAP association with change in Modified Rankin Scale (mRS).

Intentionally	Doubling initial plasma NfL	95% CI (units); *p*-value	Doubling initial plasma GFAP	95% CI (units); *p*-value
Baseline change	0.1217 units	(−0.2756, 0.5191); *p* = 0.5334	−0.1064 units	(−0.6242, 0.4114); *p* = 0.6753
Disease duration slope change	−0.05096 units/year	(−0.1993, 0.09736); *p* = 0.4963	−0.03034 units	(−0.1698, 0.1091); *p* = 0.6663

	Doubling synchronous plasma NfL (units)	95% CI (units); *p*-value	Doubling synchronous plasma GFAP (units)	95% CI (units); *p*-value
Unadjusted	0.41	(−0.01, 0.82); *p* = 0.0552	0.21	(−0.33, 0.75); *p* = 0.4305
Adjusted for time-varying age	0.47	(0.02, 0.92); *p* = 0.0404	0.31	(−0.34, 0.97); *p* = 0.3368
Adjusted for baseline age and time-varying disease duration	0.30	(−0.08, 0.67); *p* = 0.1145	0.19	(−0.33, 0.71); *p* = 0.4678

### Cytokine analysis

Plasma levels of IL-1β, IL-6, and IL-10 cytokines in LGI1 AE patients at the time of study enrollment, which is defined as below 12 months from the time of symptom onset and/or relapse, compared to non-inflammatory controls. They were analyzed using the same statistical methods as NfL and GFAP variables above, using modeling for the longitudinal analysis with back-transforming from the logarithmic to the original scale. We did not analyze longitudinal IL-6 after the enrollment of two patients participating in the double-blind study assessing satralizumab vs. placebo to avoid any concerns for unblinding within the study.

## Results

### Participant demographics

A total of 21 participants with LGI1 AE and 16 non-inflammatory controls were included. Participants in the LGI1 AE group were 76.2% male with an average age of 63.6 ± 8.6 years at the onset of symptoms, and participants in the non-inflammatory control group were 56.3% male with an average age of 58.0 ± 8.6 years. LGI1 AE patients were identified as 85.7% White and 4.8% Black/Asian/Other, and were 14.3% Hispanic.

### Clinical features

Of the LGI1 AE cohort, 90.5% presented with seizures, 85.7% with memory loss, and 38.1% with personality changes at the time of their presentation. The mean (standard deviation [SD]) diagnostic latency (time from symptoms onset to time of diagnosis) was 86.8 (86.0) days, median interquartile range [IQR] was 8 1 [1,131] days with a range from 0 to 273 days. Of those with seizures, the most common type was FBDS (57.9%), followed by focal aware and/or focal impaired aware seizures (26.3%) and generalized tonic–clonic seizures (15.8%) (Table 3). When assessing seizure activity at greater than 2 years following diagnosis, one of the 14 patients (7.1%) that had reached this time frame still had seizure activity, with 30.7% (4/14) of these patients’ seizures currently controlled on antiseizure medications (ASMs) and 64.2% (9/14) currently controlled off ASMs.

**Table 3:** Clinical data of participants with leucine-rich glioma inactivated 1 autoimmune encephalitis (LGI1 AE).

Patient ID/sex/age of onset*	Seizure type	Abnormal MRI?	Cognitive/behavioral symptoms	CSF WBC count	CSF Protein	CSF Oligoclonal bands	Initial MOCA after diagnosis (years from symptoms onset)	Initial mRS after diagnosis (years from symptoms onset)	LGI1 + in CSF?	Treatment
1/M/74	FBDS	N	Memory loss	ND	ND	ND	22 (4.0)	3 (0)	ND	Steroids IVIg, PLEX, RTX, Cellcept
2/F/55	Focal temporal lobe	Y	Memory loss	2	29	0	17 (0.50)	3 (0.48)	N	Steroids, Plex, RTX
3/M/66	Focal temporal lobe	Y	Memory loss and personality changes	4	52	ND	ND	4 (0.08)	N	Steroids, IVIg, RTX
4/M/38	No seizures	Y	Memory loss and personality changes	11	104	1	26 (0.69)	3 (0.07)	N	Steroids, IVIg, Cellcept RTX
5/M/70	FBDS	N	Memory loss and personality changes	3	61.7	ND	17 (0.76)	3 (0.28)	Y	Steroids, IVIg, PLEX, RTX
6/M/50	FBDS	Y	Memory loss	1	66	0	ND	4 (0.33)	Y	Steroids, IVIg, RTX
7/F/73	FBDS	Y	None	ND	ND	ND	ND	3 (0.30)	N	Steroids, PLEX, IVIg, RTX
8/M/61	GTC	N	Memory loss and personality changes	ND	55	0	22 (0.15)	5 (0.06)	Y	Steroids, PLEX, RTX
9/F/72	FBDS	N	Memory loss	1	29.7	ND	24 (0.26)	4 (0.07)	N	Steroids, IVIg, PLEX, RTX
10/M/63	FBDS	N	Memory loss	ND	ND	ND	26 (0.46)	3 (0.46)	N	Steroids, Cellcept
11/F/53	No seizures	N	Memory loss	ND	32	0	23 (0.23)	3 (0.23)	N	Steroids, RTX
12/M/81	FBDS	ND	Memory loss	ND	44	ND	21 (0.36)	4 (0.36)	N	Steroids IVIg, RTX
13/M/58	Focal temporal lobe	Y	Memory loss and personality changes	ND	42	0	ND	3 (0.59)	N	Steroids, PLEX, RTX
14/M/62	GTC	N	Memory loss	ND	62	0	11 (0.68)	4 (0.64)	N	Steroids, IVIg, SAT, RTX
15/M/57	FBDS	N	Memory loss and personality changes	1	39	ND	5 (0.81)	4 (0.79)	Y	Steroids, IVIg, PLEX, RTX
16/M/65	GTC	Y	Memory loss and personality changes	24	111	ND	22 (0.31)	3 (0.25)	Y	Steroids, IVIg, PLEX, SAT
17/M/83	FBDS	Y	Memory loss and personality changes	1	58	0	15 (0.25)	4 (0.21)	Y	Steroids, SAT, RTX
18/F/61	Focal temporal lobe	Y	None	ND	16	3	n/a	5 (0)	N	Steroids, PLEX, RTX
19/M/48	FBDS	N	None	ND	50	0	16 (0.10)	4 (0)	N	Steroids, SAT
20/M/63	FBDS	Y	Memory loss	ND	ND	ND	28 (1.88)	3 (0)	N	Steroids, IVIg, RTX
21/M/76	Focal temporal lobe	Y	Memory loss	300	1,154	ND	23 (1.26)	4 (0.50)	N	Steroids, IVIg, RTX

FBDS, faciobrachial dystonic seizures; F, female; GTC, generalized tonic–clonic seizure; IVIg, intravenous immunoglobulins; M, male; N, no; ND, not done; PLEX, plasma exchange; RTX, rituximab; SAT, satralizumab vs. placebo study.

**Figure 2:**
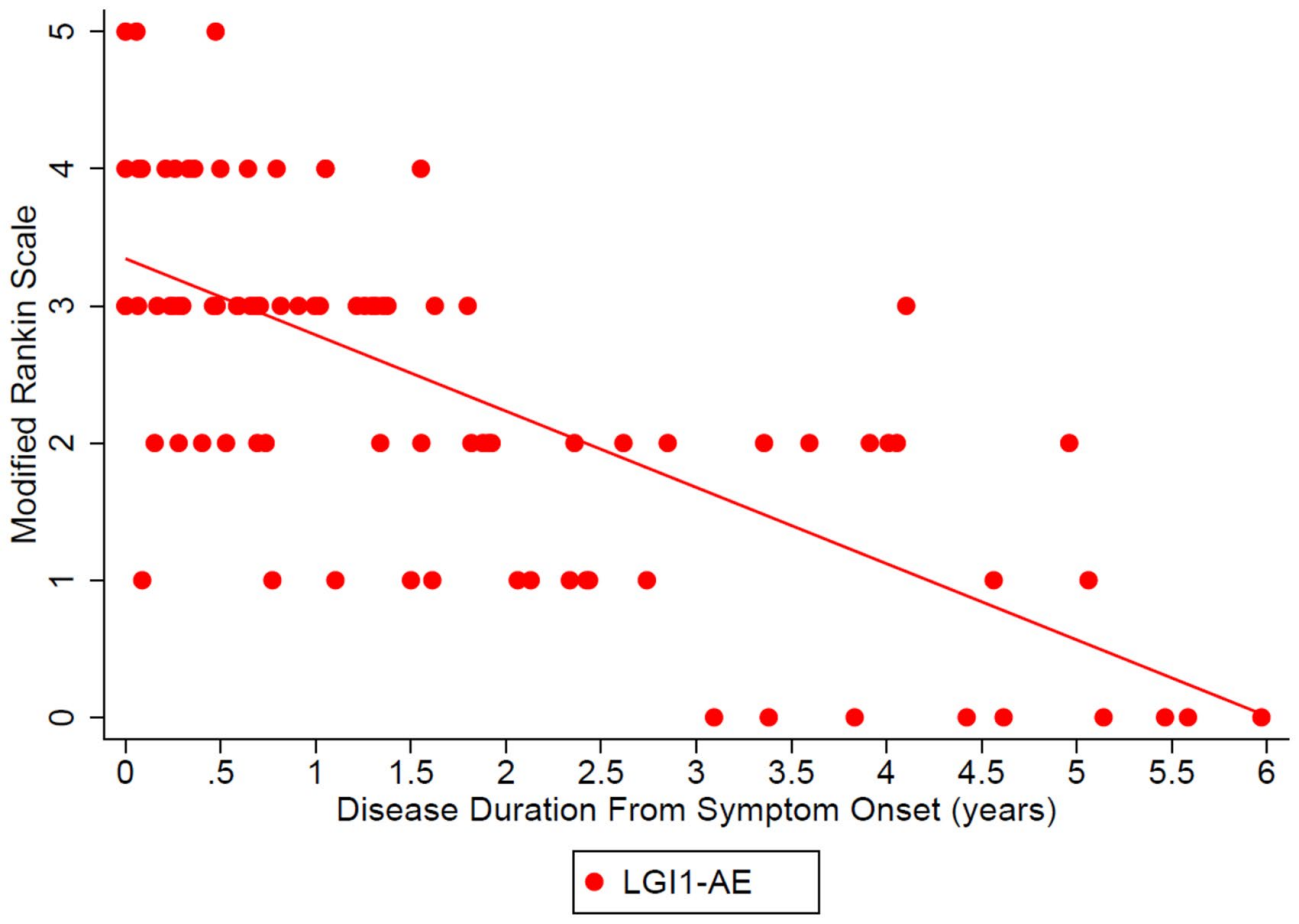
The Modified Rankin Scale (mRS) was analyzed in the LGI1 AE cohort from the time of symptom onset longitudinally up to 6 years. A linear relationship was fit with longitudinal regression, slope estimate: −0.56 per year (95% CI: (−0.68, −0.43), *p* < 0.0001). Starting age was set to the mean of the first observation of mRS in LGI1 patients, at an age of 63.6 years.

**Figure 3:**
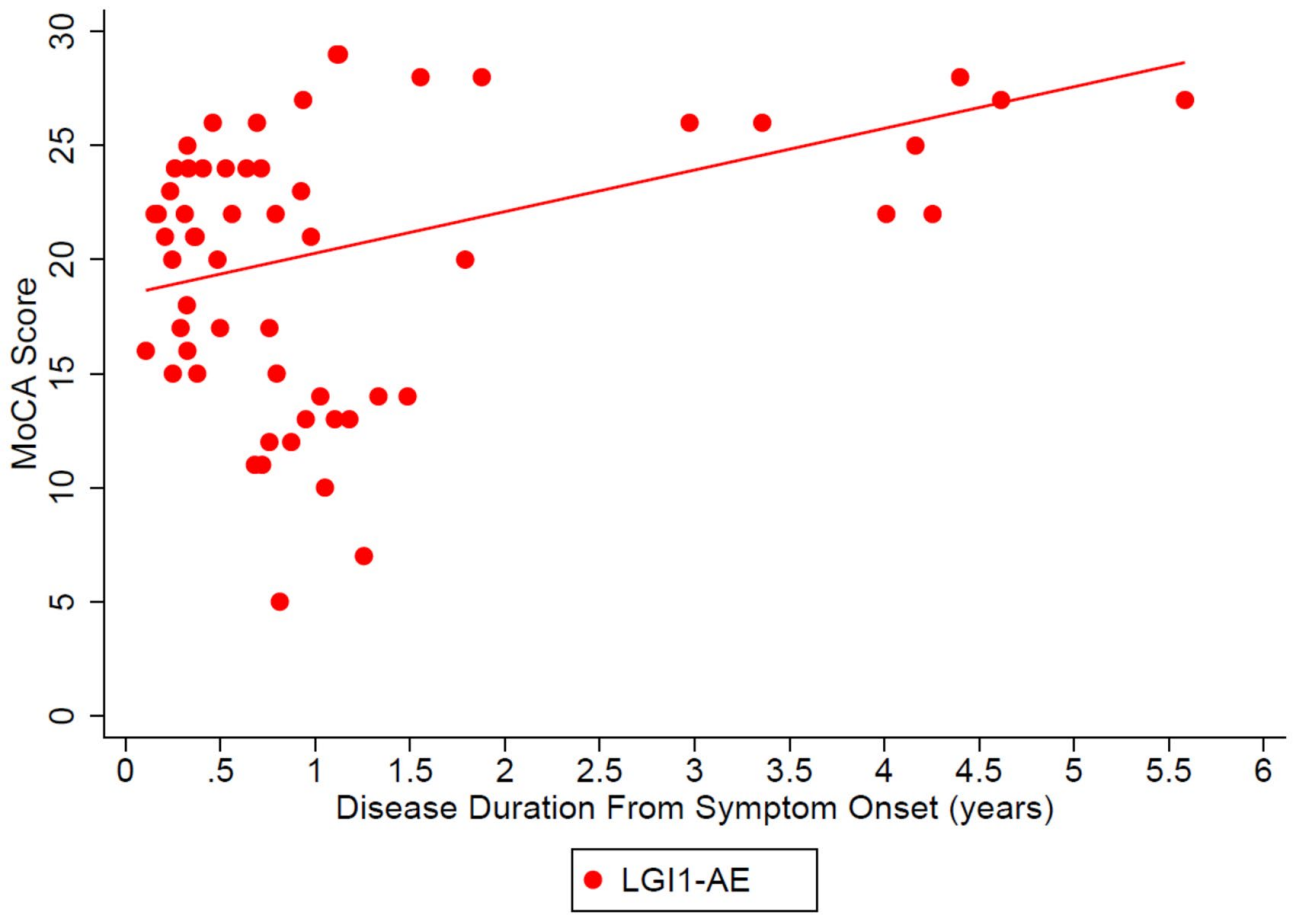
Longitudinal Montreal Cognitive Assessment (MoCA) was analyzed in the LGI1 AE cohort from the time of symptoms. A linear relationship was fit with longitudinal regression, slope estimate: 1.83 per year (95% CI: (−0.40, 4.05), *p* = 0.1006). Starting age was set to the mean age of the first observation of MoCA in LGI1 patients, 64.4 years.

The estimated mean value of the mRS at symptom onset was 3.34 and dropped to 0.56 at 5 years (*p*-value of disease duration slope was below 0.0001) (Figure 2). MoCA scores also improved (18.45 at onset, 29.40 at 6 years, *p*-value of disease duration slope = 0.1006) (Figure 3). Estimates of mean values were based on the mean ages of the first measurements of mRS and MoCA in LGI1 patients, 63.6 years for mRS and 64.4 years for MoCA.

### Laboratory testing

All LGI1 AE patients had a positive serum anti-LGI1 antibody by CBA, and 30.0% of those tested (6/20) were positive in the CSF. In the CSF, the median WBC count was 2.5 (range: 1.0–300.0) (reference: below 5 WBC/mm^3^), protein level was 52.0 (range: 16.0–1138.0) (reference: below 45 mg/dL), IgG index was 0.51 (range: 0.28–0.63) (reference: 0.25–0.70), and 11.1% (1/9) had positive unique oligoclonal bands.

### MRI features

Initial brain MRI was available for 20 out of 21 patients. Hippocampal lesions classic of limbic encephalitis (i.e., T2 hyperintensities within the mesial temporal lobe) were reported in 50% (10/20). A total of 11 patients had follow-up MRI in 1–2 years, with 4 patients showing subsequent hippocampal atrophy.

### Associated malignancy

All patients had a CT chest and abdomen scan for basic malignancy workup for paraneoplastic disease. No patients were found with thymoma or other associated malignancies.

### Immunotherapy

First-line immunotherapy consisted of 100.0% of patients receiving steroids, 61.9% receiving IVIg, and 47.6% receiving plasma exchange (PLEX). 28.6% (6/21) of the patients received steroids, IVIg, and PLEX, 19.0% (4/21) received just steroids, and 52.4% (11/21) received a combination of steroids and either IVIg or PLEX for first-line therapy. For second-line immunotherapy, 85.7% of patients received rituximab, 19.0% were enrolled in a double-blind study assessing satralizumab vs. placebo (NCT05503264), and 14.3% received mycophenolate mofetil.

### Plasma biomarkers of neuronal and glial injury

The model estimated geometric mean plasma NfL values at disease diagnosis to be 11.86 pg/mL, compared to non-inflammatory controls 6.07 pg/mL (Table 4); estimates were made based on the LGI1 patients’ first 4-plex plasma measurement at a mean age of 64.2 years, as the models were adjusted for starting age. The ratio of means of NfL in LGI1 AE vs. non-inflammatory controls was 1.55 (*p* = 0.0372) at the time of symptom onset, and LGI1 AE approached non-inflammatory controls each year for the following 6 years, but was marginally non-significant (Table 5). The model estimated geometric mean plasma GFAP values were 77.70 pg/mL at symptom onset, compared to non-inflammatory controls at 36.26 pg/mL; estimates were made at the same age as in NfL (Table 4). The ratio of means of GFAP in LGI1 AE vs. non-inflammatory controls was 1.73 (*p* = 0.0018) at the time of symptom onset and 1.50 (*p* = 0.0218) at 3 years following. LGI1 AE approached non-inflammatory controls each year for 6 years following, but without reaching statistical significance (Table 5).

**Table 4:** Mean plasma NfL and GFAP levels at symptom onset of Leucine-Rich Glioma Inactivated 1 Autoimmune Encephalitis (LGI AE) compared to non-inflammatory controls.

Diagnosis	Geometric mean NfL level (pg/mL)	Geometric mean GFAP level (pg/mL)
LGI1 AE	11.86 (CI: 8.37–16.80)	77.70 (CI: 58.32–103.51)
Non-inflammatory controls	6.07 (CI: 4.37–8.43)	36.26 (CI: 28.73–45.76)

Estimates were made using the models, with the LGI1 patients’ mean first 4plex measurement—an innovative multiplex assay that detects SARS-CoV-2, Flu A, Flu B, and Respiratory Syncytial Virus [RSV] in one test, at an age of 64.2 years.

GFAP, glial fibrillary acidic protein; NfL, neurofilament light; LGI1 AE, leucine-rich glioma inactivated 1 autoimmune encephalitis; CI, confidence interval.

**Table 5:** Plasma NfL and GFAP ratio of mean values in leucine-rich glioma inactivated 1 autoimmune encephalitis (LGI1 AE) vs. non-inflammatory controls.

Time from symptom onset (years)	NfL level in LGI1 vs. control ratio of means	GFAP in LGI1 vs. control ratio of means
0	1.55 (*p* = 0.04)*	1.73 (*p* = 0.002)*
1	1.42 (*p* = 0.07)	1.65 (*p* = 0.003)*
2	1.31 (*p* = 0.17)	1.57 (*p* = 0.007)*
3	1.20 (*p* = 0.38)	1.50 (*p* = 0.022)*
4	1.10 (*p* = 0.68)	1.42 (*p* = 0.068)

Biomarker	Percent decrease per year of disease	Percent increase per year of age
LGI1 AE NfL	8.1% (*p* = 0.14)	3.0% (*p* = 0.002)*
LGI1 AE GFAP	4.72 (*p* = 0.26)	3.0% (*p* = 0.0004)*

*Significant when *p* < 0.05.

GFAP, glial fibrillary acidic protein; NfL, neurofilament light; LGI1 AE, leucine-rich glioma inactivated 1 autoimmune encephalitis.

### Association of plasma NfL and GFAP with clinical outcome

**Figure 4:**
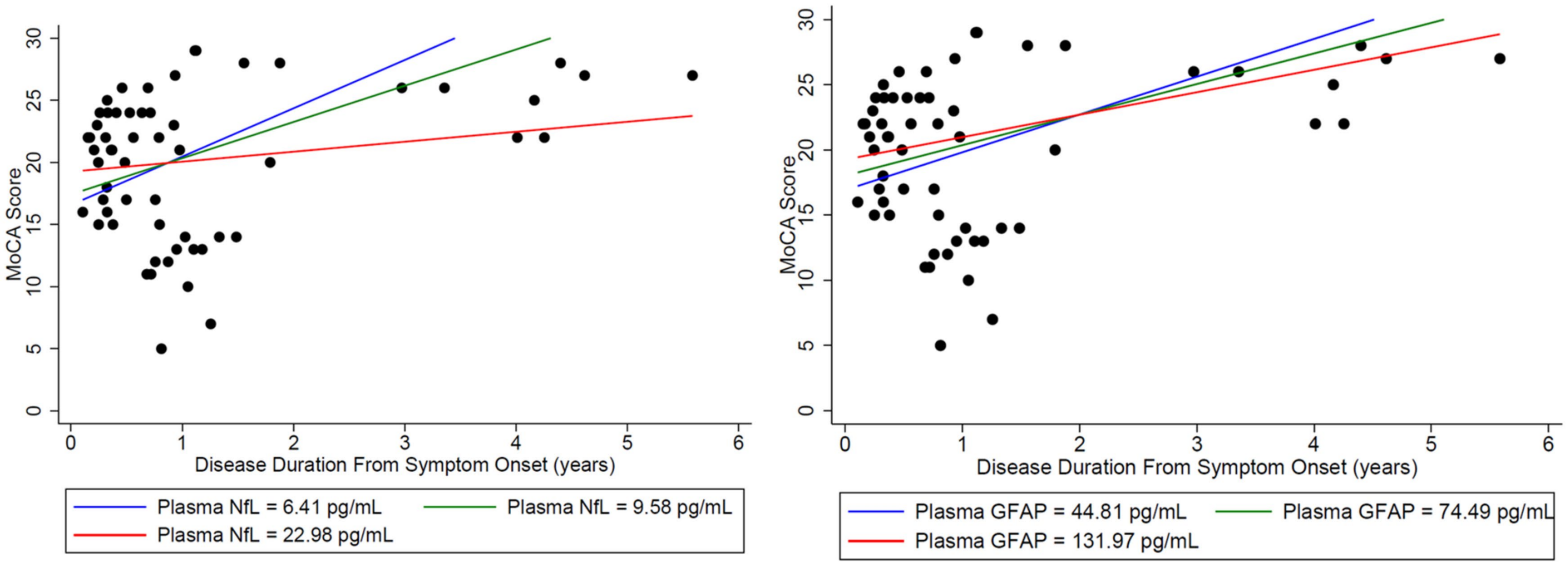
Initial NfL and GFAP levels at the onset of disease were used to analyze the estimated change in both MoCA and mRS to assess whether initial elevations in each biomarker impacted long-term outcomes in the MoCA score. Time-varying NfL and GFAP were tested for synchronous association with MoCA (closest match within 30 days). For matching NfL and GFAP to the MoCA data, there were 20 usable observations on 13 unique patients. Modeling was adjusted for age. Nfl (left) and GFAP (right) levels were broken down by quartiles using the lower quartile (blue), median (green), and upper quartile (red). A doubling of initial plasma NfL (left) was estimated to change expected MoCA at baseline by 1.4556 units (95% CI: (−2.8657, 5.7769), *p* = 0.4785), and to change the disease duration slope by −1.6760 units per year (95% CI: (−4.6996, 1.3476), *p* = 0.2611). A doubling of initial plasma GFAP (right) was estimated to change expected MoCA at baseline by 1.4981 units (95% CI: (−3.8240, 6.8201), *p* = 0.5516), and to change the disease duration slope by −0.7545 units per year (95% CI: (−3.2826, 1.7736), *p* = 0.5354). Plasma NfL in the lower quartile (blue) and median (green) trended toward a normal MoCA score of 30/30 at years 3 and 4, respectively. Patients with plasma NfL in the upper quartile (red) continued to have lower MoCA scores throughout the disease duration at 6 years. No similar trend was identified for GFAP.

**Figure 5:**
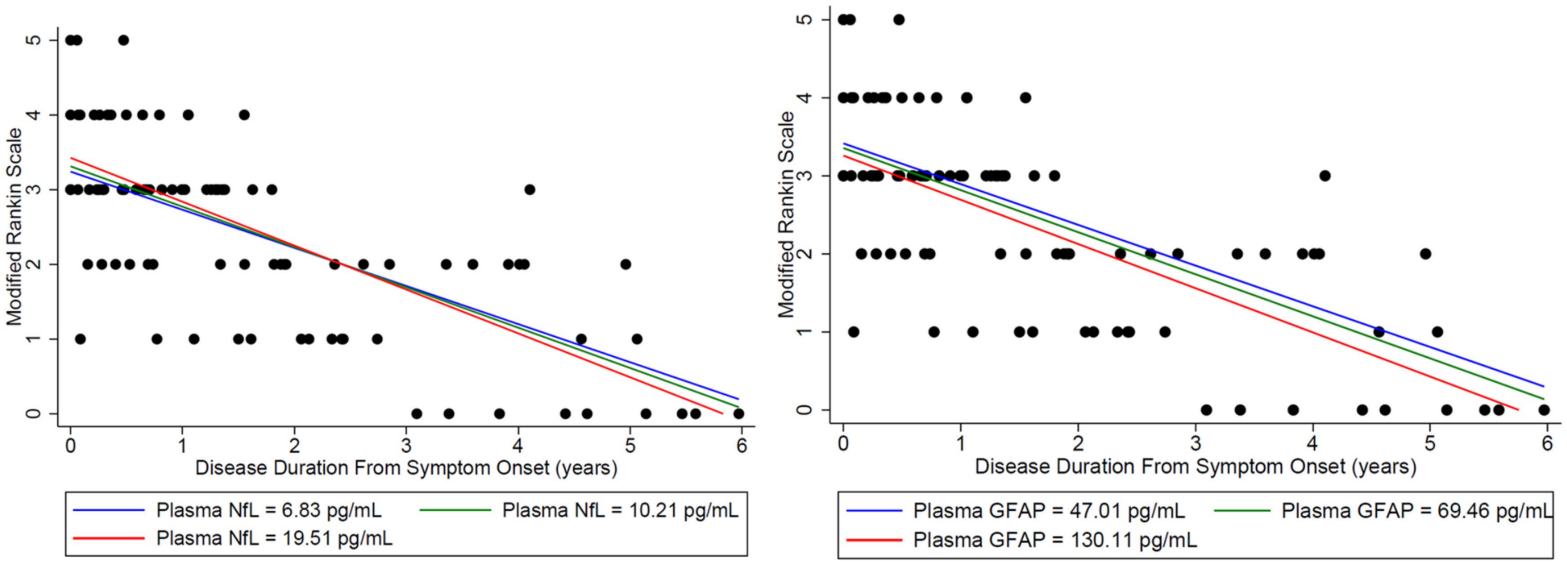
Initial NfL and GFAP levels at the onset of disease were used to analyze the estimated change in both MoCA and mRS to assess whether initial elevations in each biomarker impacted long-term outcomes in the Modified Rankin Score (mRS). Time-varying NfL and GFAP were tested for synchronous association with mRS (closest match within 30 days). To match the NfL and GFAP to the mRS data, there were 30 usable observations on 19 unique patients. Modeling was adjusted for age. Nfl (left) and GFAP (right) levels were broken down by quartiles using the lower quartile (blue), median (green), and upper quartile (red). A doubling of initial plasma NfL was estimated to change expected mRS at baseline by 0.1217 units (95% CI: (−0.2756, 0.5191), *p* = 0.5334), and to change the disease duration slope by −0.05096 units per year (95% CI: (−0.1993, 0.09736), *p* = 0.4963). A doubling of plasma initial GFAP was estimated to change expected mRS at baseline by −0.1064 units (95% CI: (−0.6242, 0.4114), *p* = 0.6753), and to change the disease duration slope by −0.03034 units per year (95% CI: (−0.1698, 0.1091), *p* = 0.6663). Patients trended toward an mRS of ≤2 regardless of initial plasma NfL and GFAP levels.

Plasma NfL in the lower quartile and median, trended toward a normal MoCA score of 30/30 at third and fourth years, respectively, and patients with plasma NfL in the upper quartile, continued to have lower MoCA scores throughout the disease duration of 6 years (Figure 4). However, when associating initial plasma NfL with MoCA, a doubling of initial plasma NfL was estimated to change expected MoCA at baseline by 1.4556 units (95% confidence interval [CI]: (−2.8657, 5.7769), *p* = 0.4785), and to change the disease duration slope by −1.6760 units per year (95% CI: [−4.6996, 1.3476], *p* = 0.2611), which was not statistically significant (Table 1). Patients trended toward an mRS of ≤2 regardless of initial plasma NfL and plasma GFAP levels (Figure 5).

### Plasma cytokine biomarkers

**Figure 6:**
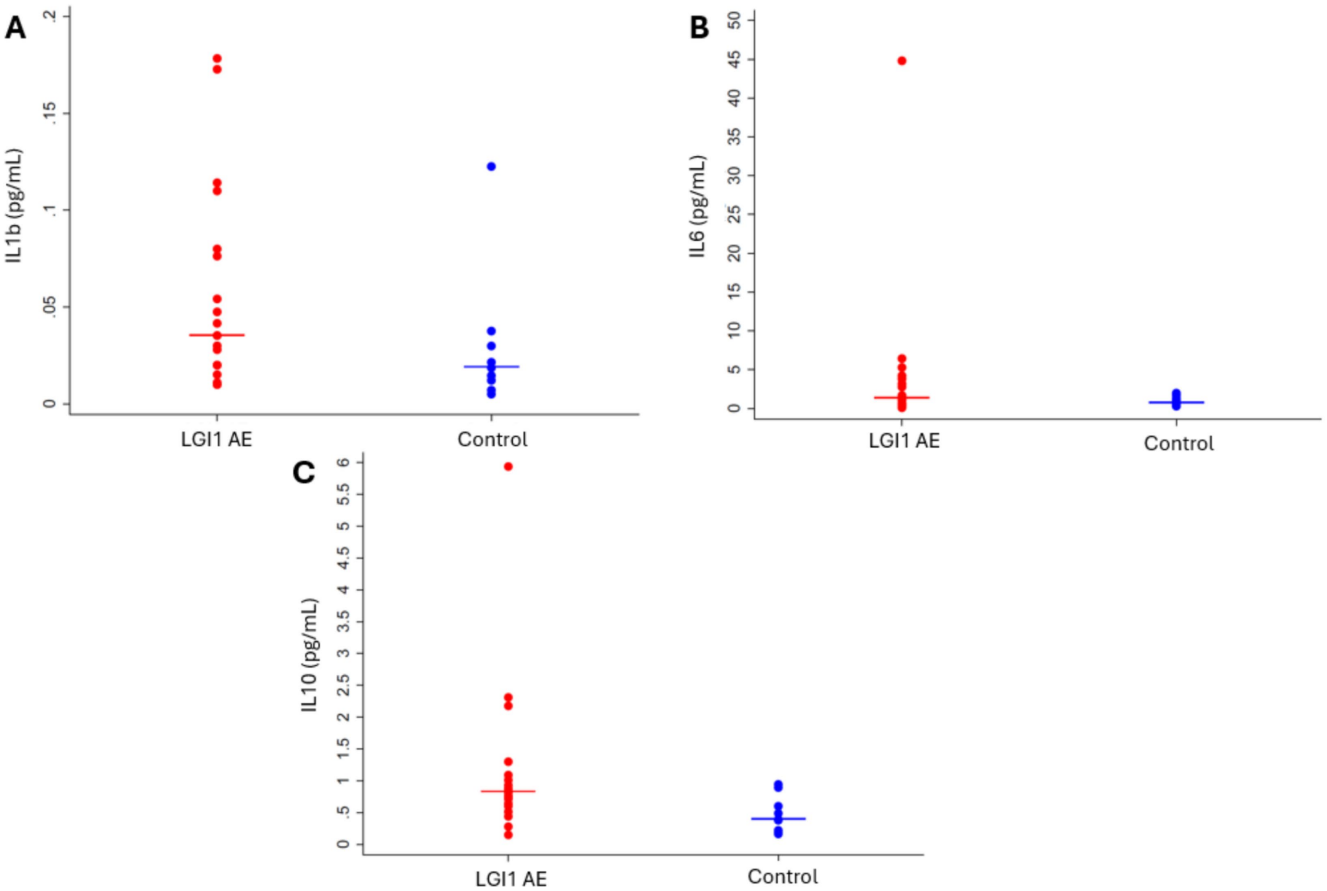
Cytokines **(A)** IL-1β (*p* = 0.1215), **(B)** IL-6 (*p* = 0.1333), and **(C)** IL-10 (*p* = 0.0134) were elevated in LGI1 AE at the time of diagnosis when analyzed cross-sectionally using the first cytokine measurement compared to controls.

Initial plasma levels of IL-1β, IL-6, and IL-10 cytokines in LGI1 AE patients compared to non-inflammatory controls showed differences, with only IL-10 being significant (Figure 6). Cytokine IL-10 showed significant variability with patient age in LGI1 AE and controls; comparisons were made at the LGI1 patients’ mean first cytokine measurement age for the available LGI1 AE patients of 63.5 years. For IL 10 in LGI1 AE, there was a ratio of geometric means of 6.97 (*p* < 0.0001) at the time of symptom onset compared to non-inflammatory controls and was still statistically significant at the following 2 years, with a ratio of geometric means of 2.80 (*p* = 0.0052) (Table 6).

**Table 6:** AE at the time of diagnosis when Plasma IL-1β and IL-10 ratio of mean values in leucine-rich glioma inactivated 1 autoimmune encephalitis (LGI1 AE) vs. non-inflammatory controls.

AE at the time of diagnosis when	AE at the time of diagnosis when	AE at the time of diagnosis when
0	2.65 (*p* = 0.037) *	6.97 (*p* < 0.0001) **
1	1.92 (*p* = 0.099)	4.42 (*p* = 0.0001) **
2	1.39 (*p* = 0.434)	2.80 (*p* = 0.005) *

Biomarker	Percent decrease per year of disease	Percent decrease per year of starting age
LGI1 IL-1β	27.5% (*p* = 0.144)	1.65% (*p* = 0.144)
LGI1 IL-10	36.6% (*p* = 0.0194) *	0.90% (*p* = 0.543)

*Significant when *p* < 0.05; **Significant when *p* < 0.001.

All values have been back-transformed from the log scale. The IL-10 model has different starting age effects for LGI1 patients and controls. The ratios presented here are for the LGI1 patients’ mean first cytokine measurement of 63.5 years.

IL, interleukin; LGI1 AE, leucine-rich glioma inactivated 1 autoimmune encephalitis.

### LGI1 AE relapse

In our cohort, we had one LGI1 AE patient who relapsed during their participation in this study, with an additional patient who entered the study at the time of their relapse, 4.5 years after their initial diagnosis. For the one LGI1 AE patient who relapsed during the study, their clinical course is summarized in Figure 1. During the relapse, there was an increase in mRS with an associated increase in NfL and GFAP plasma markers. At the time of the initial diagnosis, values for NfL and GFAP were 26.44 and 133.83 pg/mL, respectively, and decreased to 5.31 and 71.32 pg/mL, respectively, at 2 years post-diagnosis. Cytokine levels for IL-6 and IL-10 dropped from 1.68 and 2.18, respectively, to 0.95 and 0.51 at 6 months post-diagnosis. Similarly, neurological disability as measured by mRS improved throughout this time course with an initial mRS score of 4 at diagnosis, followed by improvement down to 1 after 2 years. However, at the onset of clinical relapse, 4 years post-diagnosis, the patient had a return of faciobrachial dystonic seizures. At the time of the relapse, steroids, rituximab, and lacosamide were started for treatment. The symptoms resolved within 2 months of initiation. Plasma collection 7 months after confirmed relapse demonstrated a rise in Nfl and GFAP, up to 37.21 and 103.41 pg/mL, respectively. Delays in this collection were due to a lack of in-person clinical follow-up at our center during the COVID-19 pandemic. However, this increase in plasma NFL and GFAP correlated with an increase in mRS score to 3 (Figure 1 for clinical timeline and plasma biomarker levels).

## Discussion

**Figure 7:**
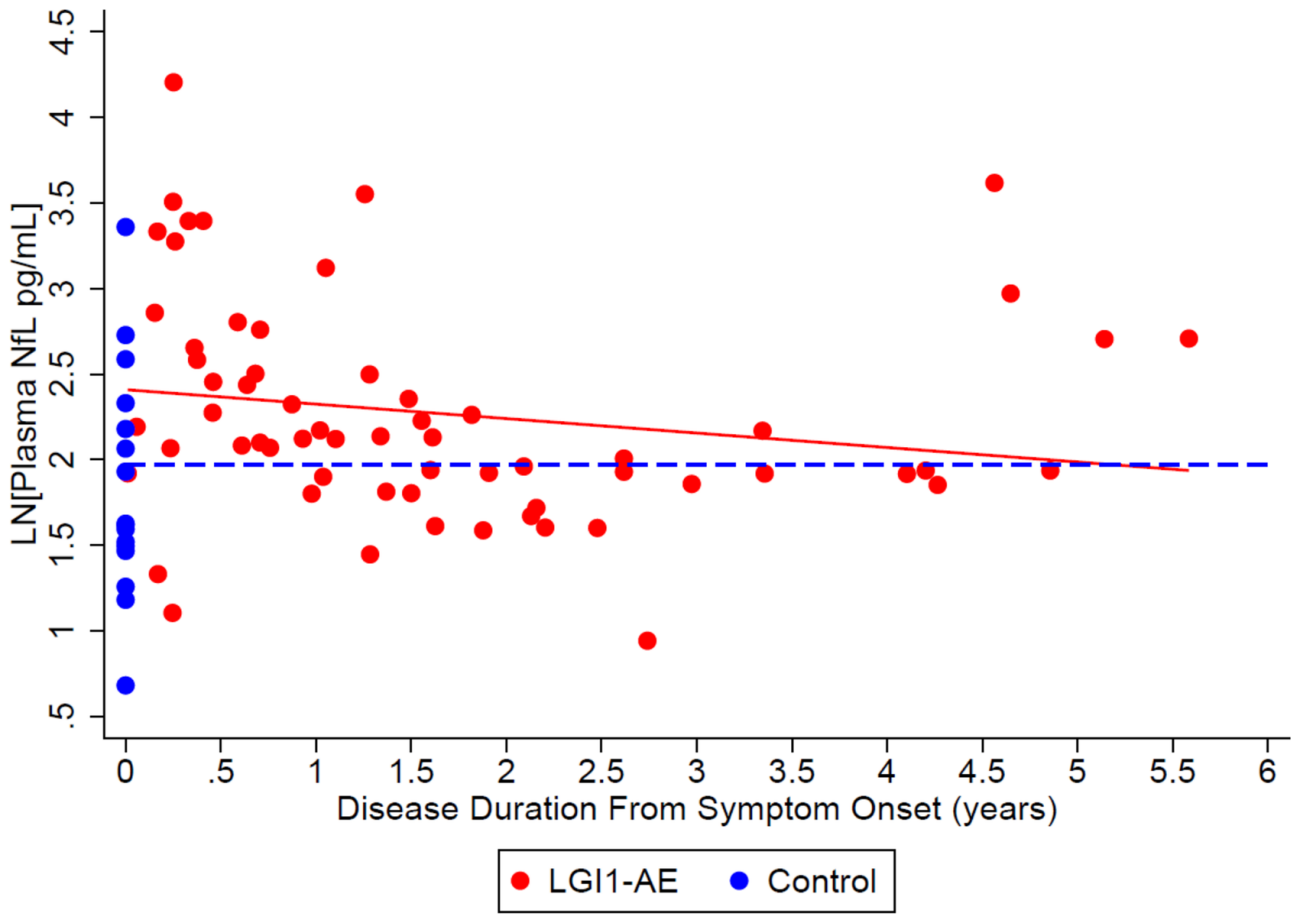
Cross-sectional analysis of 21 LGI1-AE and 16 control plasma samples showed an estimated geometric mean from regression plasma concentration of neurofilament light chain (NfL) of 11.13 pg/mL compared to non-inflammatory controls, 7.19 pg/mL. The ratio of geometric means of NfL in LGI1 AE vs. non-inflammatory controls was 1.55 (*p* = 0.0372) at symptom onset, and LGI1 AE approached non-inflammatory controls each year for 6 years. NfL decreased by 8.1% per year (*p* = 0.1404). Starting age was set to the mean of the first 4-Plex measurement at an age of 64.2 years.

**Figure 8:**
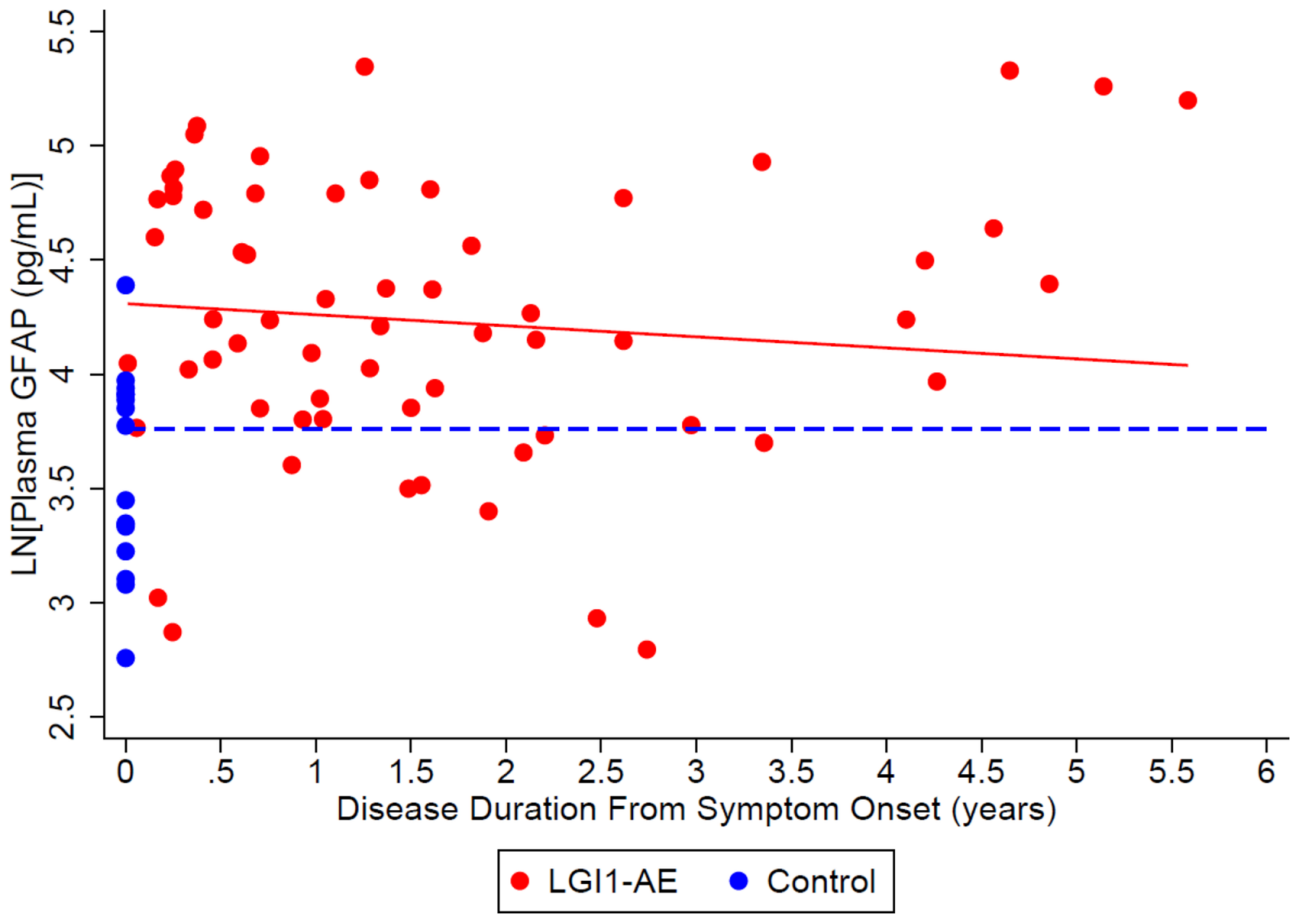
Cross-sectional analysis of 21 LGI1-AE and 16 plasma samples showed a geometric mean plasma concentration of glial fibrillary acidic protein (GFAP) of 74.36 pg/mL compared to non-inflammatory controls, 43.00 pg/mL. The ratio of geometric means of GFAP in LGI1 AE vs. non-inflammatory controls was 1.73 (*p* = 0.0018) at the time of symptom onset and 1.50 (*p* = 0.0218) at 3 years following. GFAP decreased by 4.72% per year (*p* = 0.2553).

This study demonstrates that recovery from anti-LGI1 AE continues over the years, with a decrease in mRS continuing beyond 2 years after diagnosis. At 2 years from diagnosis, all patients had an mRS of ≤ 2. Improvements in mRS continued beyond 2 years. Cognitive outcomes, measured by MoCA, improved over time as well. While not statistically significant, MoCA scores trended toward improvement up to 6 years after the onset of LGI1 AE. Overall, in this cohort, there was clinical improvement in global neurologic disability, measured by mRS, and cognitive outcomes, measured by MoCA, after standard of care treatment with immunotherapy. This trend of clinical improvement is paralleled with a slow decline in NfL and GFAP levels, returning to levels like our control population after 6 and 3 years, respectively (Figures 7, 8). Similarly, when associating initial NfL levels with MoCA scores over time, there is a trend of improvement of MoCA toward normal earlier with lower NfL levels at symptom onset (Figure 4). While not statistically significant and limited by our small sample size, this association between NfL at disease onset and cognitive outcomes may provide a useful tool for predicting cognitive recovery in LGI1 AE.

Our study has the advantage of long-term clinical follow-up up to 6 years in this rare disease. Previous studies have examined seizure freedom for up to 2 years following diagnosis (21, 22). As part of our follow-up, we assessed seizure freedom and medication use up to 6 years following symptom onset. Seizure resolution in our LGI1 cohort was common, with 30.7% of patients being seizure-free with the use of ASMs and 64.2% without using ASMs noted after at least 2 years of follow-up. This provides reassurance to patients regarding long-term prognosis, similar to prior reports in the literature (15).

The ability to correlate specific biomarkers of neuronal and glial injury and inflammatory markers like cytokines, with disease activity over time, allows for a better understanding of disease progression, with possible applications for monitoring response to immunotherapy and prognosis. Having a blood-based biomarker as a monitoring tool would provide advantages over the need for CSF monitoring. Recognizing how these plasma biomarkers change with clinical relapse and cognitive outcomes could be a useful tool in the clinic. We assessed longitudinal differences in these biomarkers and cytokines in the entire cohort, which included a patient followed throughout a clinical relapse. The mRS worsened to 3 with relapse, and the NfL and GFAP values also increased back to levels similar to the initial diagnosis at about 6 months following the relapse. This shows evidence that NfL and GFAP levels may be useful blood markers of disease and relapse activity, as they correlate with changes in disease progression.

The use of plasma cytokine levels to understand LGI1 AE is relatively unexplored, but multiple cytokines involved in inflammation pathways, including hIL-1β, hIL-6, and hIL-10, were noted to be elevated at the time of disease diagnosis, with a decline in hIL-1β and hIL-10 over time after implementation of immunotherapy. A limitation to this approach includes the variability of the use of immunotherapies in our cohort, as these cytokine levels are directly impacted by the immunotherapies used, with the majority of patients on rituximab and B cell depletion, which will lead to a direct impact on the levels of these cytokines.

Furthermore, prior reports suggest that relapses are common in LGI1 AE, with a reported relapse rate of 35 (2) and 15–25% (22) in another retrospective study. Interestingly, in our cohort followed prospectively, only one patient had an apparent clinical relapse once immunotherapy was discontinued, thus a relapse rate of 4.8% in our cohort. Our clinical practice typically uses second-line therapies, most commonly with rituximab (85.7% of patients received rituximab), with treatment over a typical course of 2 years. The patient who relapsed in our cohort received a shorter course of rituximab, which was discontinued after 1 year due to the COVID-19 pandemic. Thirty-four months after the last rituximab infusion, they had an abrupt return of FBDS consistent with a clinical relapse of LGI1 AE. Another patient who was enrolled in our prospective study at the time of LGI1 AE relapse had only received first-line therapies of steroids and IVIg since the initial diagnosis 4 years prior. There could be several factors contributing to the difference in relapse rates compared to prior reports (2, 22); however, one hypothesis may include the rapid use and prolonged course of second-line therapy. Earlier diagnosis with increased awareness of the disease could also contribute to more prompt treatment initiation. Randomized, controlled clinical trials are necessary to answer whether prolonged use of second-line therapy over 2 years has a long-term impact on the reduction of relapse rates.

Limitations of this study include small sample size, the capture of blood-based biomarkers with standard of care clinical visits, variability in clinical assessments, and variability in the timing of collection. The COVID-19 pandemic also created significant delays in collections and a decrease in in-person assessments.

## Conclusion

In our cohort, overall long-term outcomes of patients are favorable with improved neurologic disability measured by mRS, resolution of seizures, and minimal clinical relapses. While there was a trend toward improving MoCA scores, this was not statistically significant, and like other reports, ongoing mild cognitive impairment is commonly reported (1, 2). We demonstrate an initial elevation of NfL and GFAP, which down-trended, returning to similar levels of controls with improvement in the mRS and MoCA scores. It may be helpful to include these blood-based biomarkers as exploratory measures in the future randomized controlled trials.

## Data Availability

The original contributions presented in the study are included in the article/supplementary material, further inquiries can be directed to the corresponding author.
